# Determinants of Anemia among Children Aged 6–59 Months Living in Kilte Awulaelo Woreda, Northern Ethiopia

**DOI:** 10.1155/2014/245870

**Published:** 2014-09-15

**Authors:** Gebremedhin Gebreegziabiher, Belachew Etana, Daniel Niggusie

**Affiliations:** ^1^Department of Public Health, College of Health Science, Adigrat University, P.O. Box 50, Adigrat, Ethiopia; ^2^School of Public Health, College of Health Science, Mekelle University, P.O. Box 1871, Mekelle, Ethiopia

## Abstract

*Introduction.* The aim of this study was to determine the prevalence of anemia and determinant factors among children aged 6–59 months living in Kilte Awulaelo Woreda, eastern zone.* Method.* A community based cross-sectional study was conducted during February 2013 among 6 tabias of Kilte Awulaelo Woreda, northern Ethiopia. A total of 568 children were selected by systematic random sampling method. Anthropometric data and blood sample were collected. Bivariate and multivariate logistic regression analyses were performed to identify factors related to anemia.* Result.* The mean hemoglobin level was 11.48 g/dl and about 37.3% of children were anemic. Children who were aged 6–23 months [AOR = 1.89: 95% CI (1.3, 2.8)], underweight [AOR = 2.05: 95% CI (1.3, 3.3)], having MUAC less than 12 cm [AOR = 3.35: 95% CI (2.1, 5.3)], and from households with annual income below 10,000 Ethiopian birr [AOR = 4.86: 95% CI (3.2, 7.3)] were more likely to become anemic.* Conclusion.* The prevalence of anemia among the children is found to be high. It was associated with annual household income, age, and nutritional status of the child. So, improving family income and increasing awareness of the mother/caregiver were important intervention.

## 1. Introduction

Anemia can be defined as a reduction in the hemoglobin, hematocrit, or red cells number. In physiologic terms, anemia is any disorder in which the patient suffers from tissue hypoxia due to decreased oxygen carrying capacity of the blood [1]. It is mainly caused by iron deficiency in all developing countries, including Africa, where consumption of iron is limited. This is because iron-rich or animal based foods are not affordable by most families. Children <2 years and pregnant women are most at risk for anemia because their requirements for iron are higher than any other group [2].

Anemia is said to be a severe public health problem when its prevalence is 40% or more in any group (all types of anemia). Severe anemia (hemoglobin < 7 g/dL) is a public health problem if prevalence exceeds 2% [2]. According the 2004 World Health Organization (WHO) report, more than 2 billion people worldwide are anemic and about 47.4% of preschool children are affected by the problem. It affects most of countries in Africa and South Asia and some countries in East Asia and the Pacific. The highest prevalence of anemia is in Africa, but the greatest numbers of children affected are found in Asia [3].

Furthermore, according to 2008 WHO report, more than half of the world's preschool-age children (56.3%) reside in countries where anemia is a severe public health problem [3]. In sub-Saharan Africa, it is a sever public health problem among preschool-age children. In this region, much of the national prevalence is estimated to be above 40% among this group [4].

In Ethiopia, more than four out of ten under-five children (44%) were anemic. From these, about 21% of children were mildly anemic, 20% were moderately anemic, and 3% were severely anemic. In Tigray region, the reported prevalence (37.5%) was lower than the national prevalence [5].

Factors associated with anemia among children are complex and multidimensional. These involves socioeconomic, nutritional, biological, environmental, and cultural characteristics [6]. Because of this, understanding these factors in a given population is important for evidence based interventions and policies towards anemia. Many researches have been conducted to show its associated factors. But it remains the main public health problem, especially in developing countries. So, identifying factors associated with anemia is needed to develop appropriate interventions.

Therefore, the study was designed to assess the prevalence of anemia and its associated factors among children aged 6–59 months living in Kilte Awulaelo Woreda, eastern zone, northern Ethiopia. So it provides evidences for policy makers and program managers for policy formulation, problem prioritization, and resource allocation.

## 2. Methods 

### 2.1. Study Area and Population

The study was conducted during February 2013 in Kilte Awulaelo Woreda which is located in eastern zone of Tigray regional state. It is found 828 km north of the capital city, Addis Ababa. There are 115,762 people within 25,047 households in the Woreda (source: Woreda Kilte Awulaelo Plan and Finance Office, 2012). It is divided into 18 tabias (the smallest administration unit in Tigray) and the main source of income is agriculture. The Woreda has 5 health centers and 16 health posts that provide health services. The main causes of morbidity among children in the Woreda were pneumonia, other respiratory infections, and malnutrition.

### 2.2. Study Design and Sample

Community based cross-sectional study was conducted among 568 children and their mothers/caregivers. The sample size was calculated using single population proportion formula assuming the prevalence of all types of anemia among children 6–59 months of age in Tigray regional state is 37.5% [7], confidence interval 95%, margin of error 5%, and design effect 1.5. Finally, 5% of the calculated sample size added for any non response to the actual sample size.

Multistage sampling was used to select study participants. For this, first, 6 tabias were selected using lottery method from 18 tabias in the Woreda. Within these tabias, households with children aged 6–59 months were identified after census was conducted and the number of households was allocated by proportional allocation to size method. Finally, individual households were selected by systematic random sampling technique after sampling fraction was prepared for each tabia separately. In case of more than one child within the specified age group within the household, one child was selected by lottery method.

### 2.3. Data Collection

Data were collected by interviewing mother/caregivers of the child during house to house survey using pretested and interview administered questionnaire which was prepared in English language which was later translated into Tigrigna. Three types of data from respondents were children and their caretaker/mother characteristics, anthropometric data, and blood sample from children. Diploma graduate clinical nurse interviewed mothers/caregivers and anthropometric data were taken by the three health extension workers. In addition, three laboratory technicians collected blood sample.

One-day training was given for data collectors and supervisors before data collection which was followed by pretesting of the tool. The pretesting was done in one tabia which was not included in the study. The data collection team completed a total of 30 questionnaires during pretest and necessary changes were made accordingly.

Height and length board was used to take height/length of children and mothers. Recumbent length was taken for children aged 6–23 months and standing height was taken from children aged 24–59 months. In addition to this, mid-upper arm circumference was taken by MUAC tape. Furthermore, weighing scale was used to take weight measurements for all children and mothers. Known weight (premeasured) was used daily in the morning and afternoon to check the quality of the weighing scale. To maintain accuracy of anthropometric measurements, anthropometric data was collected twice from children and mothers and the average of the two measurements was taken.

Blood sample was collected using diamine tetraacetic acid (EDTA) test tube and transported to Wukro Hospital every day to measure the hemoglobin level. Laboratory technician did the hemoglobin test using complete blood count (CBC) machine in Wukro Hospital Laboratory Department. To protect the quality of collected blood sample, it was transported using icebox to nearby hospital (Wukro Hospital) within eight hours after collection.

The quality of hemoglobin test was further insured by continuously washing the machine by cleaning solution (cell clean) and measuring blank solution/air/background until it is near to zero (<0.1 g/dL) reading. The machine washing was done every day by cleaning solution until the background/air reading reaches near to zero. In addition to this, the quality of test was also checked by using standard eight checks which are commercially prepared and have known hemoglobin value. After analysis, the samples were discarded to the sink in the laboratory and the test tubes were incinerated in incinerator.

### 2.4. Measurement

Anemia was defined as presence of hemoglobin level of less than 11 g/dL. It was further categorized into mild, moderate, and severe anemia. Mild anemia was for child with hemoglobin level of 10–<11 g/dL, while moderate anemia was for children with Hb level of 7–<10 g/dL and severe anemia was for hemoglobin level below 7 g/dL.

Besides this, the children national status was assessed and classified into underweight, stunting, and wasted. Children are said to be underweight if they had weight-for-age *z*-score below −2 standard deviation; stunting was for children with height-for-age *z*-score below −2 SD while wasted was for those with weight-for-height *z*-score below −2 SD according to WHO Child Growth Standards median.

### 2.5. Data Analysis

Data was entered and cleaned using Epi-Info 3.5.1 and analyzed using SPSS 16. The data was cleaned and preliminary analysis was done by the researchers. Descriptive statistics was done to describe the data. Then, binary logistic regression was made to see the crude associations between independent variables and dependent variable. Variables found to have statistically significant association, *P* value < 0.05, during bivariate analysis were entered to multiple logistic regressions to identify independent predictors of anemia after controlling for confounder. Odds ratios (OR) with their 95% confidence level (CI) were calculated. Anthropometric data was analyzed using ENA software using 2006 WHO standards [8]. All statistical tests are considered significant at *P* < 0.05 level.

Hemoglobin level less than 11 mg/dL was taken as dependent variable while household income, mothers' characteristics, child characteristic, source of water and availability of toilet facility, antenatal care (ANC) visit, child feeding, and child nutritional status were taken as independent variables.

### 2.6. Ethical Statement

An ethical approval was obtained from the Ethical Review Committee of Mekelle University, College of Health Sciences, Research and Community Service Office. Official support letter was obtained from Mekelle University, Tigray Regional Health Bureau, and Kilte Awulaelo Health Office for conducting the study. Information about objective of the study, procedures, potential risks, and benefits was given to mothers before they were enrolled to the study.

All participants were informed about the purpose and significance of the study before their consent was taken. Their full right to refuse participation was explained. Written informed consent was obtained from each mother/caregiver of children selected for the study. Children found with severe anemia (Hb value of 7–<11 g/dL) got free treatment from the health post and were counselled to visit nearby health facility for further investigation and treatment. In addition to this, referral paper was provided to take the child to the nearby health facility. There is no potential risk occurring to participants except minimal discomfort while blood was drawn from the children. So, the right of participants to anonymity and confidentiality will be ensured by making the questionnaire anonymous.

## 3. Results

### 3.1. Characteristics of Study Participants

Total of 568 households participated in the study with the 100% response rate. The mean age of mothers was 28.34 (±6.46) years which ranged from 19 to 51 years. Majority (95%) of the respondents were Orthodox followers while 13 (2.3%) were Muslims. In addition, the majority, 510 (89.8%), of the respondents were married and 264 (46.5%) of the mothers had formal education (they can read and write). Majority of the mothers, 485 (85.4%), were housewives and 83 (14.6%) of them had their own income with mean annual income of 13195 (Table 1).

Agriculture was the main source of income for majority of the respondents (510 (89.8%)) and the remaining 23 (4%) were employed. More than half of the households, 337 (59.3%), use pipe water (hand pipe) and the remaining 329 (40.7%) use water from other sources like river water and well. Majority of the households, 456 (80.3%), owned latrine/toilet and the remaining 112 (19.7%) have no latrine (Table 1).

### 3.2. Child Characteristics

From total of 568 selected children, 282 (49.6%) of them were males and 286 (50.4%) were females. The mean age of the children was 30.18 (15.85) months. Beside this, about 52.4% were stunted and 6.5% were wasted, while 20.6% were underweight. On average (±SD), the child ate 2.87 (0.79) food groups per day (Table 2).

On average, there are about 3.46 siblings per household and the mean birth interval between the selected child and his/her elder was 32.8 months.

### 3.3. Magnitude of Anemia among Children

Anemia was measured using the hemoglobin level of the child. So, accordingly, the mean hemoglobin level was about 11.48 (±1.53) g/dL which ranged from 5.5 g/dL to 14.5 g/dL. Accordingly, more than one-third of the children, 212 (37.3%), were anemic and only 2 (0.4%) of them were found to be severely anemic, whereas 65 (11.4%) were moderately anemic and 145 (25.5%) were mildly anemic. The prevalence of anemia was 40.2% among females and 34.5% among males (Table 3).

**Figure 1:**
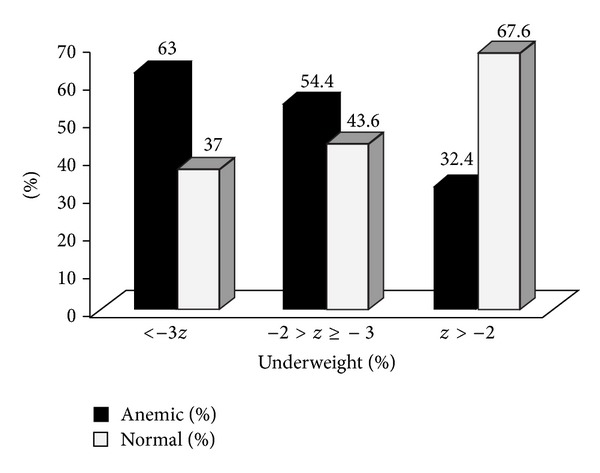
Anemia among children aged 6–59 months by their nutritional status in Kilte Awulaelo Woreda, northern Ethiopia, during February 2013.

Figure 1 shows anemia by weight-for-age (underweight) *z*-score. Anemia was higher among children who are severely underweight and it is lower among children with normal weight-to-age *z*-score.

**Figure 2:**
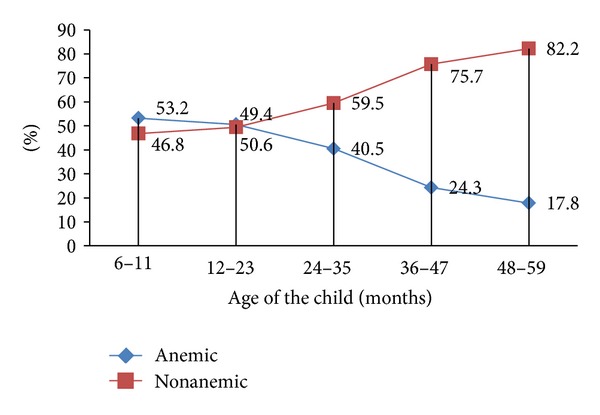
Anemia among children aged 6–59 months by their age distribution in Kilte Awulaelo Woreda.

**Figure 3:**
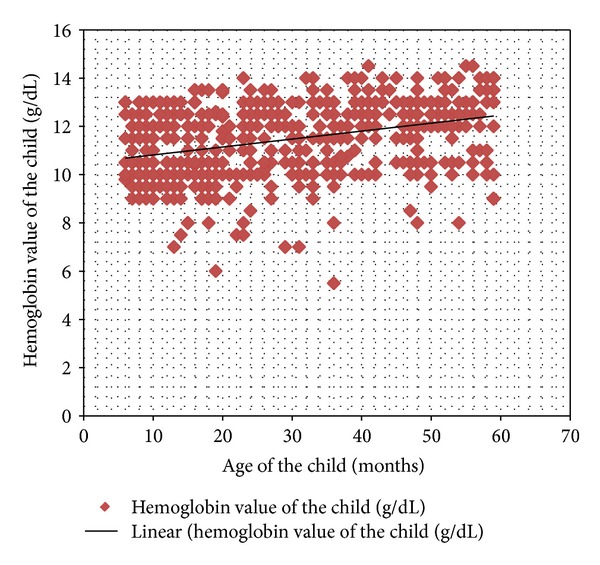
Hemoglobin value of the children aged 6–59 months in relation to their age, Kilte Awulaelo Woreda, eastern zone, northern Ethiopia, 2013 (*n* = 568).

Furthermore, it also differs by child age category. Younger children were more anemic than older children and it gradually decreases as the child gets older. Children aged 6–23 months were the most at-risk group in which the risk is almost 3 times when compared with those of 48–59 months (Figures 2 and 3).

### 3.4. Factors Associated with Anemia

Bivariate logistic regression analysis was done to assess association of sociodemographic and other maternal factors with child anemia (Table 4). Accordingly, children of mothers with weight of less than 50 kg were 1.77 (1.2, 2.6) times more likely to be anemic than those of mothers greater than 50 kg. But it did not show statistically significant association in multivariate logistic regression. The remaining maternal factors did not show statistically significant association with child anemia even in the bivariate logistic regression (Table 5).

Table 6 shows the association of household factor with anemia among children. Multivariate logistic regression analysis showed that children from household with annual income of less than 10,000 birr were 4.86 [COR, 4.86; 95% CI: (3.2, 7.3)] times more likely to be anemic than their counterparts. The remaining household factors did not show statistically significant association with child anemia in both bivariate and multivariate logistic regression.

Other factors assessed to be associated with child anemia were child age and dietary factors. Multivariate analysis showed that children aged 6–23 months were 1.89 (1.2, 2.8) times more likely to be anemic than those of 24–59 months of age. Furthermore, anemia also related to nutritional status of the children. Being underweight, that is, with weight-for-age less than −2 *z*-score, 2.05 (1.3, 3.3), and having mid-upper arm circumference (MUAC) less than 12 cm [3.35 (2.1, 5.3)] were significantly associated with anemia (Table 6).

## 4. Discussion

This study assessed the prevalence of anemia and factors associated with it among children aged 6–59 months. The prevalence of anemia was found to be 37.3% which is relatively lower than the national prevalence (44%) of the EDHS 2011 findings, but it is similar to the prevalence of Tigray region (37.5%) [9]. Prevalence of anemia reported from several developing countries varied. It is about 16.1% in the Philippines during the year 2008 [10] and 87% in Tanzania [11]. This level of prevalence is considered moderate public health problem according to WHO classification [2], but it is lower than the estimated global anemia prevalence (47.4%) [3].

We found that the prevalence decreased with age. It is dramatically decreased among children aged above 23 months. This finding is similar to study conducted in Nigeria in 2011 [12]. This may be attributable to lower iron requirements per kg body weight associated with decreasing growth rate and the shift in diet from complementary foods to table foods. This is supported with studies that have found that children under two years of age groups were more anemic than children aged 2–5 years [10, 12–16]. The first 2 years of life carry the highest risk for developing anemia [17, 18]. Iron requirements are related to growth velocity and so requirement per kg of body weight decreases with age. The prevalence of the problem in under-24-month-old children is likely to be a combined result of the increased iron requirements due to rapid growth, low availability of foods rich in iron, and lack of diet variety. Iron intake is also likely to improve with age as a result of a more varied diet, including the introduction of meat and other iron containing foods [13].

In addition to this, age of the child had statistically significant association with anemia using multivariate logistic regression. Children aged 6–11 months were the most affected age groups with anemia prevalence of 53.2% which is almost three times higher than those aged 48–59 months (17.8%). This finding is similar to study findings done in Brazil (2010), Bangladesh (2010), and northern Ethiopia (2007) [8, 16, 19].

In this study, anemia among children was also associated with household income. Children living in household with lower monthly income were more likely to have anemia compared to those with higher income. Similar finding was from study conducted in Brazil (2011 and 2010) and in northern Ethiopia in 2007 [8, 16, 19]. This is due to the reason that children from poor households are less likely to get iron-rich foods like animal foods and vitamin-rich foods especially vitamins A and C which are very important for iron absorption. In addition to these, households were less likely to afford health service during illness. But, study conducted in the state of Pernambuco, Brazil, in 2007 shows no association between household income and child anemia [15].

Nutritional status of the children also related to anemia among children aged 6–59 months. In this study, malnourished children were more likely to be anemic than well-nourished children. Children who were underweight and have MUAC less than 12 cm were more likely to be anemic than their counterparts. This finding is supported by findings from Brazil and Tanzania [11, 20]. Since anemia and malnutrition often share common causes, it is expected that multiple nutrition problems would cooccur in the same individuals [8]. These factors are aggravated by poverty and food insecurity [19]. Low intake of iron-rich foods and diminished nutrient absorption caused by changes in the gastrointestinal epithelium in malnourished individuals contribute towards development of anemia [16].

However, mother and child sociodemographic characteristics are not shown to be associated with anemia among children under five. The influence of child sex on anemia shows no association in this study. This finding was also supported with studies conducted in Lao People's Democratic Republic in 2011 and Morocco in 2010 [18, 21]. But this is contrary to study done in the Philippines which reported that anemia is more common in male children [10]. Besides this, maternal education and employment status were also not associated with anemia among children aged 6–59 months old. This finding is supported by study conducted in Brazil in 2011 [16]. This may be due to the reason that most of the mothers that were included in the study were illiterate and most of those that attend formal school were at elementary school level. Because of this, our sample is not sufficient to ascertain statistical association.

In this study, numbers of children in the household, birth interval, water supply, and availability of latrine were not associated with presence of anemia among children aged 6–59 months. This result was supported with reports of two studies conducted in Brazil (2011 and 2010) for number of children in the household, water source, and presence of latrine [13, 20]. But study conducted in the Philippines shows an association between anemia and water supply [19]. Availability of latrine was also not associated with anemia in this study. Besides this, our result showed that maternal BMI was not associated with anemia among children aged 6–59 months. Contradictory result was found from study conducted by WHO in Brazil and India [16, 22].

This study has used the best available technology (Cysmex machine) to determine hemoglobin level which has more accuracy and this makes it strong. But it has certain limitations. Most of the questions asked like dietary recall, ANC visit, and age of the child and the mother may be subject to recall bias. Besides this, this study can only show the local prevalence of anemia and cannot show temporal relationship between anemia and other factors considered. Given these limitations, our finding has a great contribution to improving the health of children aged 6–59 months.

## 5. Conclusion 

The prevalence of anemia among children aged 6–59 months was a moderate public health problem with 37.3% according to WHO classification. Annual income below 10,000 Ethiopian birr, age of the child between 6 and 23 months of age, and being underweight (children with WAZ less than −2 *z*-score and MUAC of children less than 12 cm) were predictors of anemia among children aged 6–59 months.

So, policy makers should focus on activities that can improve household income to ensure sufficient food production. Moreover, interventions like iron supplementation and nutritional education activities are important to decrease the prevalence of anemia. Finally, controlling multiple nutritional deficiencies among children by expanding targeted supplementary feeding programs, at health post level, which targets malnourished children, is mandatory to avert other nutritional problems including anemia.

## Figures and Tables

**Table 1:** Sociodemographic and other characteristics of the mother in Kilte Awulaelo Woreda, eastern zone, northern Ethiopia, 2013 (*n* = 568).

Variables	Frequency	Percent
Marital status		
Married	510	89.8
Single	24	4.2
Divorced	29	5.1
Widowed	5	0.9
Religion		
Orthodox	553	97.4
Muslim	13	2.2
Catholic	2	0.4
Educational status		
No formal education	305	53.7
1–6	162	28.5
7-8	46	8.1
9–12	46	8.1
Above 12	9	1.6
Earned annual income		
Yes	83	14.6
No	485	85.4
ANC visit		
None	38	6.7
1 time	59	10.4
2 times	175	30.8
3 times	216	38
4 times and more	80	14.1
Annual income of the HH		
≤5000	3	0.5
5001–10000	189	33.3
10001–15000	218	38.3
15001–20000	134	23.6
20001–25000	18	3.2
>25001	6	1.1
Availability of latrine/toilet		
Yes	456	80.3
No	112	19.7
Source of income for the HH		
Agriculture	510	89.8
Employed	23	4
Merchant	27	4.8
Daily laborer	8	1.8

**Table 2:** Characteristics of child and dietary factors in Kilte Awulaelo Woreda, eastern zone, northern Ethiopia, 2013 (*n* = 568).

Variable	Frequency	Percentage
Age of the child in months		
6–23	231	40.7
24–35	116	20.4
36–47	103	18.1
48–59	118	20.8
Mean age (±SD)	**30.18 (15.85)**	
Sex of the child		
Male	282	49.6
Female	286	50.4
MUAC in cm		
<12	37	6.5
≥12	531	93.5
Stunted		
Yes	298	52.4
No	270	47.6
Underweight		
Yes	117	20.6
No	451	79.4
Wasted		
Yes	37	6.5
No	531	93.5
Number of food groups eaten by the child per day		
≤3	465	81.8
≥4	103	18.2
Mean food groups (±SD)	**2.87 (0.79)**	

**Table 3:** The degree of anemia among children aged 6–59 months in relation to their sex in Kilte Awulaelo Woreda, northern Ethiopia, 2013 (*n* = 568).

Hemoglobin value	Male (%)	Female (%)	Total *N* (%)
<7 g/dl (severely anemic)	0 (0)	2 (0.7)	2 (0.4)
7–9.9 g/dl (moderately anemic)	33 (11.2)	32 (11.2)	65 (11.4)
10–11.9 g/dl (mildly anemic)	64 (22.7)	81 (28.3)	145 (25.5)
≥11 g/dl (normal)	185 (65.5)	171 (59.8)	356 (62.7)

**Table 4:** Association of sociodemographic and other maternal factors with anemia in children aged 6–59 months in Kilte Awulaelo Woreda, eastern zone, northern Ethiopia, 2013 (*n* = 568).

Variable	Anemic		Odds ratio (95% CI)	
	Yes (%)	No (%)	COR (95% CI)	OR (95% CI)
Maternal factors				
Age of mother				
<30 years	126 (36.3)	217 (63.3)	0.94 (0.6, 1.3)	
≥30 years	86 (38.2)	139 (61.8)	1	
Maternal weight				
<50 kg	133 (44.3)	167 (55.7)	**1.77 (1.2, **2.6)*	2.27 (0.4, 13.7)
≥50 kg	55 (31.1)	122 (68.9)	1	
Religion of the mother				
Orthodox	207 (37.4)	346 (62.6)	1	
Others	5 (33.3)	10 (67)	0.84 (0.28, 2.48)	
Marital status				
Married	185 (36.2)	326 (63.8)	0.79 (0.35, 1.77)	
Divorced	9 (31)	20 (69)	0.28 (0.09, 0.82)	
Others	18 (62)	11 (38)	1	
Employment status of the mother				
Employed	34 (41)	49 (59)	1.20 (0.7, 1.9)	
Not employed	178 (36.7)	307 (63.3)	1	
Mother's ability to read and write				
Yes	99 (37.6)	164 (62.4)	1.03 (0.7, 1.4)	
No	113 (37)	192 (63)	1	
ANC visit				
Yes	13 (34.2)	25 (65.8)	0.86 (0.4, 1.7)	
No	199 (37.5)	331 (62.5)	1	
Maternal BMI				
Underweight	39 (41.5)	55 (58.5)	0.53 (0.1, 2.5)	
Normal	145 (38.6)	231 (61.4)	0.47 (0.1, 2.1)	
Overweight	4 (57.1)	3 (42.9)	1	

*Variables show statistically significant association at *P* value < 0.05. OR: odds ratio.

**Table 5:** Association of household factors with anemia in children aged 6–59 months in Kilte Awulaelo Woreda, eastern zone, northern Ethiopia, 2013 (*n* = 568).

Variables	Anemic		Odds ratio (95% CI)	
	Yes (%)	No (%)	COR (95% CI)	AOR (95% CI)
Household factors				
Annual income				
<10000	87 (68.5)	40 (31.5)	**5.50 (3.6, **8.4)*	**4.86 (3.2, **7.3)*
≥10000	125 (28.3)	316 (71.7)	1	
Availability of latrine/toilet				
Yes	162 (35.5)	294 (64.5)	0.68 (0.4, 1.0)	
No	50 (44.5)	62 (55.4)	1	
Availability of pipe water (hand)				
Yes	120 (35.6)	217 (64.4)	0.84 (0.6, 1.2)	
No	92 (39.8)	139 (60.2)	1	
Source of income for the household				
Agriculture	188 (36.9)	322 (63.1)	0.97 (0.2, 4.1)	
Employed	5 (21.7)	18 (78.3)	0.46 (0.1, 2.6)	
Merchant	16 (59.3)	11 (40.7)	2.42 (0.5, 12.3)	
Daily laborer	3 (37.5)	5 (62.5)	1	

*Variables show statistically significant association at *P* value < 0.05. OR: odds ratio.

**Table 6:** Association of child factors with anemia in children aged 6–59 months in Kilte Awulaelo Woreda, eastern zone, northern Ethiopia, 2013 (*n* = 568).

Variable	Anemic		OR (95% CI)	
	Yes	No	Crude OR (95% CI)	Adjusted OR (95% CI)
	Number (%)	Number (%)		
Child factors				
Age				
<24 months	119 (51.5)	112 (48.5)	**2.79 (1.9, **3.1)*	**1.89 (1.2, **2.8)*
≥24 months	93 (27.6)	244 (72.4)	1	
Sex of the child				
Male	97 (34.4)	185 (65.6)	0.78 (0.5, 1.1)	
Female	114 (40.0)	171 (60)	1	
MUAC of the child				
<12 cm	30 (81.1)	7 (18.9)	**8.22 (3.5, **19.1)*	**3.35 (2.1, **5.3)*
≥12 cm	182 (34)	349 (65.7)	1	
Birth order of the child				
≤3	133 (37.3)	224 (62.7)	0.99 (0.7, 1.4)	
≥4	79 (37.4)	132 (62.6)	1	
Birth interval of the child				
≤33 months	16 (32.7)	33 (67.3)	1	
>33 months	11 (15.1)	62 (84.9)	**0.41 (0.1, **0.9)*	0.42 (0.1, 1.3)
Number of children in household				
≤3 children	128 (37.5)	213 (62.5)	1.02 (0.7, 1.4)	
≥4 children	84 (37)	143 (63)	1	
Child wears shoe				
Yes	184 (36.2)	324 (63.8)	0.65 (0.4, 1.1)	
No	28 (46.7)	32 (53.3)	1	
Number of food groups eaten by the child				
≤3 food group	81 (45)	99 (55)	1	
≥4 food group	131 (33.8)	257 (66.2)	**0.32 (0.2, **0.5)*	0.26 (0.04, 1.5)
Stunting				
Yes	129 (43.3)	169 (56.7)	**1.72 (1.2, **2.4)*	2.85 (0.8, 10.3)
No	83 (30.7)	187 (69.3)	1	
Underweight				
Yes	66 (56.4)	51 (43.6)	**2.70 (1.8, **4.1)*	**2.05 (1.3, **3.3)*
No	146 (32.4)	305 (67.6)	1	
Wasting				
Yes	23 (60.5)	15 (39.5)	**2.76 (1.410, **5.430)*	3.06 (0.8, 10.5)
No	189 (35.7)	341 (64.3)	1	

*Variables show statistically significant association at *P* value < 0.05. OR: odds ratio.
